# Effects of music learning and piano practice on cognitive function, mood and quality of life in older adults

**DOI:** 10.3389/fpsyg.2013.00810

**Published:** 2013-11-01

**Authors:** Sofia Seinfeld, Heidi Figueroa, Jordi Ortiz-Gil, Maria V. Sanchez-Vives

**Affiliations:** ^1^Department of Systems Neuroscience, Institut d'Investigacions Biomèdiques August Pi i SunyerBarcelona, Spain; ^2^Franz Liszt Music SchoolBarcelona, Spain; ^3^Psychology Unit, Hospital General de Granollers, FIDMAG, CIBERSAMBarcelona, Spain; ^4^Institució Catalana Recerca i Estudis AvançatsBarcelona, Spain; ^5^Department of Basic Psychology, Universidad de BarcelonaBarcelona, Spain

**Keywords:** music, piano, brain plasticity, aging, elderly, training, cognitive function, quality of life

## Abstract

Reading music and playing a musical instrument is a complex activity that comprises motor and multisensory (auditory, visual, and somatosensory) integration in a unique way. Music has also a well-known impact on the emotional state, while it can be a motivating activity. For those reasons, musical training has become a useful framework to study brain plasticity. Our aim was to study the specific effects of musical training vs. the effects of other leisure activities in elderly people. With that purpose we evaluated the impact of piano training on cognitive function, mood and quality of life (QOL) in older adults. A group of participants that received piano lessons and did daily training for 4-month (*n* = 13) was compared to an age-matched control group (*n* = 16) that participated in other types of leisure activities (physical exercise, computer lessons, painting lessons, among other). An exhaustive assessment that included neuropsychological tests as well as mood and QOL questionnaires was carried out before starting the piano program and immediately after finishing (4 months later) in the two groups. We found a significant improvement on the piano training group on the Stroop test that measures executive function, inhibitory control and divided attention. Furthermore, a trend indicating an enhancement of visual scanning and motor ability was also found (Trial Making Test part A). Finally, in our study piano lessons decreased depression, induced positive mood states, and improved the psychological and physical QOL of the elderly. Our results suggest that playing piano and learning to read music can be a useful intervention in older adults to promote cognitive reserve (CR) and improve subjective well-being.

## Introduction

Decrease in fertility rates and growth in life expectancy have resulted in a dramatic increase of elderly people worldwide. It is estimated that the proportion of people over 60 years old will increase from 10% in 2000 to 21.8% in 2050 in all regions (Lutz et al., 2008). A critical consequence of this rise will be the exponential increase in the prevalence of neurodegenerative diseases and other pathologies common in latter stages of life (Norton et al., 2013). Within this context, the study of strategies to prevent cognitive decline and promote a healthy physical and psychological lifestyle are keystones for the future.

Certain deteriorations in cognitive function are triggered by normal aging and have a significant impact on the lives of elderly people (for a review see Bishop et al., 2010). White matter degeneration in frontal lobes in older adults is associated with attenuated performance in executive function, speed of processing, and memory (Gunning-Dixon and Raz, 2000; Ziegler et al., 2010). Additionally, old adults suffer age-related difficulties in motor abilities (Mattay et al., 2002). Another possible explanation for the decline might be a decreased coordination between large-scale brain systems that are subservient to higher order cognitive functions in the elderly (Andrews-Hanna et al., 2007). However, the aging brain can also initiate compensatory processes to mitigate cognitive decline. For example, there is evidence that older adults show less lateralized activity in the prefrontal cortex during performance of different cognitive tasks (Cabeza, 2002).

In this context, Cognitive Reserve (CR) has become a key concept for the prevention of neurodegenerative diseases and age-related cognitive decline. CR models postulate that the brain actively copes with brain damage by using preexisting cognitive resources or by activating compensatory mechanisms (for reviews see Stern, 2002, 2009). This notion seems to be supported by the observations of cohort studies where individuals had never manifested cognitive impairment during their lives in spite of having advanced Alzheimer's neuropathology discovered at postmortem (Ince, 2001).

Factors that have been shown to contribute significantly in increasing CR and reducing the risk of suffering dementia are educational attainment, occupational achievements, intellectual ability, social interactions, and leisure activities (for a review see Valenzuela and Sachdev, 2006). Specifically, it has been found that late-life cognitive activities (e.g., reading, writing, crossword puzzles, board or card games, group discussions, playing music, among others) may influence CR reducing the onset of accelerated memory decline by 0.18 years in subjects who develop dementia, controlling for educational level (Hall et al., 2009). Furthermore, Akbaraly et al. (2009) carried out a 4-year follow-up study of a large elderly sample and found that the engagement in stimulating activities (at least twice per week) resulted in a 50% reduced risk of developing dementia. Further studies have also corroborated the importance of engaging in leisure activities and having an active cognitive lifestyle for the prevention of cognitive decline (Verghese et al., 2003; Gow et al., 2012; Marioni et al., 2012).

Music is one of the most effective sources of stimulation in the auditory cortex and other brain areas. Listening to music generates wide-spread cortical activity that expands beyond the auditory cortex involving brain areas related to attention, semantic processing, memory, motor function, and emotional processing (Särkämö et al., 2008). Moreover, playing a musical instrument is a complex and motivating activity that comprises the coordination of multiple sensory modalities (auditory, visual, and somatosensory) and motor system in a unique way. In this sense, learning to play the piano implies acquiring the skill of musical sight-reading to translate notations into movement patterns on a keyboard. As Stewart et al. (2003) has shown, learning to read musical notation can have very specific effects on a behavioral (specific spatial mapping skills) and brain level (functional changes in the superior parietal cortex and fusiform gyrus). Therefore, music training has become a useful framework to study brain plasticity throughout the lifespan (for a review see Jäncke, 2009; Herholz and Zatorre, 2012).

A series of brain imaging studies have revealed structural brain differences between musicians and non-musicians. Professional musicians have been found to have greater than average gray matter in motor, auditory, and visuospatial areas, differences in white matter architecture, stronger asymmetry of the planum temporale, and increased corpus callosum (Schlaug et al., 1995; Schlaug, 1995; Schmithorst and Wilke, 2002; Gaser and Schlaug, 2003). These anatomical differences are thought to be due to long-term acquisition and training of musical skills. Furthermore, it has been demonstrated that there is a strong correlation between high musical activity during the lifespan and preservation of non-verbal memory, naming, and executive function (Hanna-Pladdy and MacKay, 2011). This effect seems to be mediated by the number of years involved in active musical training.

However, due to the correlational nature of the studies cited in the previous paragraph, no causal relations can be established between musical training, cognitive enhancement, and the anatomical differences in the brain. Other variables, such as congenital predispositions, age, education, and socioeconomic status, could account for the results. Therefore, the most compelling evidence of the effects of musical training is from longitudinal studies on child populations. Schellenberg (2004) found that children who received 36-weeks musical lessons (standard keyboard or Kodálay) showed a small but significant increase in IQ compared to children who took drama lessons or no lessons at all. Furthermore, it has been shown that 6-year-old children who received 15 months private keyboard lessons showed structural brain changes that correlated with improvements in musically relevant auditory and motor skills when compared to a control group that did not received such instruction (Hyde et al., 2009). Finally, a series of follow-up studies comparing 8-year-old children who received either music or painting lessons for several months, found that music training has transfer effects to linguistic abilities evidenced by improvements in behavioral measures and electrophysiological responses, while painting lessons do not (Moreno et al., 2009; Chobert et al., 2012; François et al., 2013). These studies show that there is a clear transfer effect from musical training to auditory and speech skills (for a review see Kraus and Chandrasekaran, 2010; Besson et al., 2011). Interestingly, due to the random assignment of participants to the groups and the longitudinal methodology used in these studies, the results can only be explained by music training effects and not by pre-existing predispositions.

Lappe et al. (2008) showed that even short-term musical training in adults can induce cortical plasticity. In their study, a group of adults learned to play a piano sequence, while the control group just listened to the music and judged it. Their results indicated that the group that actively played piano had an enlargement of Mismatch Negativity Potentials after training, while passive listeners did not show such a pattern. In a further study the same authors proved that including a rhythm-focused exercise in piano lessons induced a more robust plastic change (Lappe et al., 2011). Moreover, the benefits of musical training have also been shown in a stroke patient evidenced by a change in the reorganization of the sensorimotor cortex and an improvement in his movement quality after receiving music-supported therapy (Rojo et al., 2011; Rodriguez-Fornells et al., 2012). Evidence suggests that brain plasticity can also be activated in elderly populations as a result of certain training regimes. Boyke et al. (2008) demonstrated that plasticity in gray matter in the middle temporal area of the visual cortex can occur after 3 months learning juggling by elderly participants. Cortical reorganization was though stronger in young adults. This evidence raises the question of whether such cortical changes can occur as a consequence of learning a musical instrument in later stages of life.

Some studies suggest that musical training in the older stages of life can mitigate effects of the aging brain (for a review see Wan and Schlaug, 2010). Verghese et al. (2003), in a follow-up study of elderly people, observed that those individuals who played a musical instrument were less likely to suffer dementia than participants involved in other type of leisure activities like reading, writing, or doing crossword puzzles. Surprisingly, in this study physical activity was not associated with a lower risk of suffering dementia. However, there is compelling evidence that physical activity might delay the onset of dementia (for a review see Hamer and Chida, 2009). Moreover, it has been observed that age-related delays in neural timing and auditory decline can be mitigated by musical training (Parbery-Clark et al., 2011, 2012). Finally, 6 months of individualized piano lessons in older adults improved executive functioning and working memory (Bugos et al., 2007), although not all cognitive benefits were maintained in a 3-month follow-up assessment.

The impact of music on mood and quality of life (QOL) of older adults has also been researched. Depression disorders have a high prevalence in later stages of life and most of the times they are underdiagnosed and undertreated (Kiosses, 2013). In a meta-analysis, Luppa et al. (2012) indicated that pooled prevalence of major depression in older adults was about 7.2% for major depression and around 17.1% for depressive disorders. When facing depression, musical activities, such as listening and making music, can have a positive impact on the QOL and well-being of older adults by promoting empowerment, autonomy, and social cohesion, among other (Laukka, 2007; Lee et al., 2010; Solé et al., 2010; Creech et al., 2013). Moreover, active and passive musical activities in elderly people have been found to improve mood and reduce depression symptoms (Chan et al., 2010; Erkkilä et al., 2011).

In aging societies like ours it is important to understand whether learning a musical instrument in older age might act as a protective factor against cognitive decline and also in what manner it can promote subjective well-being specifically in elderly people. As has been noted above, past evidence suggests that musical training as a multimodal activity can promote brain plasticity, prevent cognitive decline and improve psychological health. Nevertheless, the conclusions that can be extracted from these studies concerning the effects of musical training in latter stages of life are limited. This is due to the fact that the vast majority of studies had a correlational nature and included samples of older adults who already had extensive experience in playing a musical instrument. Additionally, investigations that followed a longitudinal approach mainly studied samples of children or young adults.

This investigation aims to study the impact of a 4-month group piano training program that included as its principal components learning musical theory, sight-reading and playing a keyboard. Importantly, it was designed to be delivered to an elderly population (60–85 years old). In order to assess whether any improvement was specifically due to the piano lessons, a control group formed by older adults who participated in other type of leisure activities was included in the study. The program was based on a weekly lesson, complemented by 45 min of individual practice every day. To our knowledge, only the study of Bugos et al. (2007) used a similar approach although there were differences in the design since the duration of the training program was longer (6 months), the participants of their control group did not participate in other types of leisure activities, and only depression was assessed and no other emotional aspects such as mood states and QOL. Our goal was to assess changes in cognitive function, motor coordination, and emotional state as a result of the music training in elderly population to define a paradigm for future brain function studies and to further study musical training regimes that can contribute to successful aging.

## Methods

### Participants

Forty-one healthy male and female participants, 60–84 years old, took part in this study. The inclusion criteria were: being older than 60 years, naïve to reading music or playing a musical instrument and with no history of neurological seizures. Subjects were previously screened with the Mini Mental State Examination (MMSE; Folstein et al., 1975) and Frontal Assessment Battery (FAB; Dubois et al., 2000) to discard any possible mild cognitive impairment or dementia. Subjects with a score <24 in the MMSE and <14 on the FAB were excluded from the study. Moreover, the Word Accentuation Test (WAT; Del Ser et al., 1997; Gomar et al., 2011) was used to assess possible significant differences in estimated intelligence of participants. Participants were excluded if they had a current diagnosis of a neurological or psychiatric disorder that could affect cognition or require the intake of psychoactive medications.

The assignment of participants to the piano group was done upon motivation, level of interest for the activity, time available for practice and fulfillment of the inclusion and exclusion criteria. Once the piano group was complete, we recruited participants for the control group with the following requirements: matching age and level-of-education, fulfilling of the inclusion and exclusion criteria, and being involved in other leisure activities for the 4-month that the study lasted.

Twenty-five participants were assigned to the piano group, from which nine withdrew for different reasons (e.g., lack of time to practice, medical interventions, or unforeseen travel). Additionally, three participants were also excluded from the analysis due to changes in medical prescriptions that included the intake of psychoactive medications. Therefore, the final sample size consisted of 13 participants in the experimental group (nine females and four males). On the other hand, the control group included 16 participants who participated in different leisure activities (thirteen females and three males). All of the subjects in the control group practiced physical exercise. However, it should be noted that 62% of the participants practiced more than one single physical activity per week and 83% also participated in other types of academic and art training that did not include physical activity (e.g., painting, philosophy, computer, and English lessons). Table 1 summarizes in percentages the specific leisure activities that the control group practiced during the study.

**Table 1:** **Summary of leisure activities practiced by the control group**.

**Leisure activities practiced by control group**	**% of subjects involved in activity**
Workout (gym)	31.25
Cycling	12.50
Painting lessons	18.75
Excursions/long-walks	56.25
Computer lessons	18.75
Swimming	25
Dance	18.75
Pilates	12.50
Language lessons	12.50
Yoga	6.25
Tai Chi	6.25
Golf	6.25
Philosophy lessons	31.25

Due to the age of the participants, all subjects took at least one medication for the prevention of cardiovascular diseases and arthritis.

### Recruitment and consent

Participants were recruited from local community centers in the city of Barcelona. Advertising was done through posters and talks about the piano training program we offered. Piano lessons were totally free, but a requirement for participation was a high interest in the activity and time available for practice. Additionally, both control and experimental groups were asked to sign a consent form and offered a report of their neuropsychological assessment after completion of the study. The study was approved by the Ethical Committee for Clinical Research of Hospital Clinic of Barcelona.

### Psychological assessment

The measures that formed part of this study were decided with the collaboration of a professional neuropsychologist with long experience in the assessment of elderly population (Jordi Ortiz-Gil). Furthermore, before the start of the study the person in charge of running all tests (Sofia Seinfeld) was exhaustively trained to ensure that the neuropsychological assessments were administered in a standardized and neutral way. The person in charge of administering the tests and questionnaires that formed part of the study was an author of this paper not blinded to membership of the experimental and control groups.

All participants in the piano group were tested 2 weeks before the start of the piano training program and up to 2 weeks after the last piano lesson. Participants of the control group were tested 2 weeks after the piano program started and 4 months later (±2 weeks). Consequently, the time length between the pre-test and post-test was controlled for both groups. Each of the two neuropsychological evaluations that formed part of the study were carried out in a single session each lasting around one hour and a half. We believe that this is an adequate duration for the assessment since Uttl et al. (2000) found that no fatigue effects were observed in critical test in older adults undergoing a neuropsychological assessment of up to 3 h. Importantly, all tests and questionnaires were given in the same order to avoid possible order effect differences between the two groups. The evaluation battery consisted of three main blocks: in the first block demographic information was collected and screening tests were administered; in the second block all cognitive and motor test were applied; finally, mood and QOL questionnaires were completed. The specific test and questionnaires that formed part of the neuropsychological assessment are detailed below.

Subjects were questioned for age and years of education. The type of medications that participants were prescribed were also registered before and after the program implementation. Furthermore, participants of the control group self-assessed the leisure activities that they practiced during the 4 months. Finally, subjects of both groups estimated the frequency they practiced per week (in days per week).

#### Screening tests

The MMSE (Folstein et al., 1975) was used for the detection of possible moderate to severe cognitive deficits. It is a standardized test widely used as a brief screening of dementia or cognitive impairment by assessing a set of cognitive functions simply and quickly. The maximum points that a person can obtain in this inventory is 30. It has been adapted and translated for Spanish population with a good test-retest reliability and validity (Lobo et al., 1999). The cut-off point for detecting impairment in Spanish population is fixed in a score of 23/24, with a sensibility of 89.8% and specificity of 75.1%, after correcting for age and education.

The FAB (Dubois et al., 2000) was used as a brief assessment tool that has shown good validity, inter-rater reliability, and sensitivity to detect predominant dysexecutive syndrome and possible frontotemporal dementia (Slachevsky et al., 2004). FAB is composed of six subtests that explore conceptualization, item generation, motor sequencing, interference sensitivity, inhibitory control, and environmental autonomy. This battery has been translated into Spanish and tested in Spanish population, showing good psychometric properties (Rodríguez et al., 2003). The maximum score a person can obtain is 18. The cut-off point for the detection of possible frontotemporal dementia has been fixed between 13 and 12, with 89% accuracy.

The WAT (Del Ser et al., 1997) was used to calculate estimated intelligence of participants. This test is an adaptation of the North American Adults Reading Test (NART; Blair and Spreen, 1989) for Spanish speaking population. Since pronunciation of Spanish words can be derived from their spelling, the WAT utilizes low-frequency Spanish words whose accents have been removed to make pronunciation ambiguous. A recent study has shown that WAT gives a reliable IQ estimate in healthy adults (Gomar et al., 2011).

#### Motor and cognitive function

The *Finger Tapping Test* (FTT; Halstead, 1947; Reitan and Wolfson, 2009) is the most widely used test to measure manual dexterity (Lezak, 2012). This test consists of a device that contains a tapping key and a mechanical counter to record the number of taps given in an interval of time. In our assessment, subjects had to make five 10-s taps with the Right Hand (RH) and the Left Hand (LH) independently. The five trails were then averaged to get a single measure of performance.

The *Grooved Pegboard* (Klove, 1963) is a test that measures complex motor coordination and manual dexterity. For its application a board containing 5 × 5 sets of slotted holes angled in different directions was used. Subjects were instructed to insert in each of the holes a peg that has a ridged along one side. If the peg is not positioned correctly toward the slotted holes they cannot be introduced. The score is obtained from the time to complete the task with both hands independently.

*Block Design* forms part of the Spanish version of WAIS-III (Weschler, 2002) and was applied to assess visuospatial organization. The test entails the presentation of nine red and white blocks that participants must use to construct replicas of a model design presented by the examiner in a certain time period (Lezak, 2012). The score is obtained from the number of replicas that the subjects can construct in a given time (for simple model design 60 s and for complex model design 120 s).

*Digits Span Forward* (DSF) and *Digits Spain Backwards* (DSB), taken from the Spanish version of Wechsler Adults Intelligence Scale, 3rd Edition (WAIS-III; Weschler, 2002) were administered for assessing verbal immediate memory and verbal working memory, respectively. In DSF the participant has to verbally recall a sequence of numbers exactly as they were given by the examiner. Task difficulty gradually increases by having to remember each time longer sequences. The test finishes once the participant has failed to repeat correctly both attempts of the same sequence. DSF and DSB are administered similarly, except for the fact that in DSB the subject is instructed to repeat a sequence of numbers exactly in the reverse order. Studies have shown that while DSF measures attention efficiency, DSB is a more complex task related to executive function, since a mental double-tracking task that implies memory and a reversing operation simultaneously has to be made (Lezak, 2012). The amplitudes of the recalled sequences were also taken into account for the analysis.

The *Spatial Span Forward* (SSF) and *Spatial Span Backwards* (SSB; Milner, 1971) test were used for evaluating immediate non-verbal memory and non-verbal working memory, respectively. A board included in the Wechsler Memory Scale, 3rd Ed. (WMS-III; Wechsler, 2004) containing nine cubes fastened in random order was used. In SSF the participant must tap the blocks in the same order as the examiner has done in a prearranged sequence immediately before. In contrast, in the SSB the participants are asked to repeat the sequence of taps exactly in the reverse order. The test increases in difficulty as the number of taps included in a sequence increases. The test finishes once the participant has failed to repeat correctly both attempts of the same sequence. The amplitudes of the recalled sequences were also taken into account for the analysis.

The *Trail Making Test part A* (TMT-A) and *B* (TMT-B) form part of the Halstead–Reitan Battery (Reitan and Wolfson, 2009). TMT was used to assess visuomotor tracking, divided attention, cognitive flexibility, and motor function (Lezak, 2012). TMT-A consist of drawing lines as quickly as possible to link consecutively numbered circles. In contrast, in TMT-B participants must connect consecutively numbered and lettered circles by alternating between the two sequences. The scoring of TMT derives from the number of seconds to complete the task and errors are also counted. The less time it takes to complete the task, the better the performance in this test. A recent Spanish normative study showed that TMT is influenced by age and education (Peña-Casanova et al., 2009a).

The *Symbol Digit Modalities Test* (SDMT; Smith, 2002) was used to measure divided attention, visual scanning, visual tracking, perceptual speed, motor speed, and memory (Peña-Casanova et al., 2009a). The test contains a coding key that pairs nine symbols with numbers. Subjects must fill-in as many as possible of the 110 blank spaces that contain a key symbol on the top, within a 90-s interval. The scoring is obtained by counting the number of correct answers. A highest amount of correct numbers paired with their corresponding symbol in the given time interval is considered to reflect a better performance in this test.

The Spanish version of the *Stroop Test* (Golden, 1999) was used in this study. There are three conditions in this test: one implies reading black colored names of colors (Stroop-Word; SW); the second condition implies naming of the colors in which four consecutive crosses are printed (Stroop-Color; SC); and in the latter condition the examinee is expected to say, in each item, the color of ink used for printing a color name, which has been printed in a color different to the one written (Stroop Color-Word; SCW). This test is based on the finding that it takes longer to name colored symbols than to name words that refer to colors, and even longer to name the color of ink in which incongruent color names are written (Lezak, 2012). The final score for each condition is obtained by counting the number of colors correctly named or read (depending on condition) in a 45 s time interval, with higher scores indicating better performance. Stroop is a reliable measure of executive function that requires cognitive flexibility, selective attention, cognitive inhibition, and information processing speed (Peña-Casanova et al., 2009b).

The *Formal Lexical Task* (Peña-Casanova et al., 2009c) was used to assess subject's ability to generate problem solving strategies. This test consists in asking subjects to name as many words as possible starting in letters P, M, or R in a 60 s interval. A higher number of different words named in the given time interval is considered to reflect a better performance in the task. It has been shown that subjects that can figure out a strategy for guiding the search of words perform better than person that do not use strategies (Lezak, 2012). A different letter was used for each neuropsychological assessment and the order of letter presentation was randomized between subjects.

#### Mood and quality of life

The *Beck Depression Inventory* (BDI; Beck et al., 1961) is a 21-item self-report inventory commonly used in order to measure the severity of depression in adults. Responders have to answer the questionnaire according to their mood during the two previous weeks. This inventory has been translated and adapted for Spanish population with good validity and reliability (Sanz and Vázquez, 1998). In this study, BDI has been used as a screening tool for possible depression symptoms and for detecting affective changes induced by the piano intervention. Higher scores in this questionnaire indicate a higher depression severity.

The *Profile of Mood States* (POMS; McNair et al., 1971) is a questionnaire that measures fluctuations of affective mood states. Specifically, it measures six identifiable mood states: (1) Tension, (2) Depression, (3) Vigor, (4) Fatigue, (5) Anger, and (6) Confusion. POMS is a good measurement to assess acute effects of a treatment or intervention. In this study, we have used a Spanish adaptation of POMS (Balaguer, 1993) to assess possible affective changes in mood induced by piano lessons, since it has shown good psychometric properties. This version of POMS consists of 58 items composed by five-point Likert-type scale. Higher scores in this questionnaire indicate more psychological distress, except in the vigor scale that is reversed.

The *World Health Organization Quality of Life Brief Questionnaire* (WHOQOL-BREF; Kuyken et al., 1995) is a cross-cultural assessment tool consisting of 26-items extracted from the original WHOQOL-100 questionnaire. The WHOQOL-BREF uses five-point Likert-type scales to measure four main domains of QOL: (1) Physical health, (2) Psychological health, (3) Social relations, and (4) Environment health. The time frame for the assessment is the 2 previous weeks. Higher scores in this questionnaire indicate a better QOL. The Spanish version of WHOQOL-BREF was used in this study since it has shown to be a good measurement of QOL in Spanish older adults showing good consistency, validity and reliability (Lucas Carrasco, 1998; Lucas-Carrasco et al., 2011). Higher scores in each domain of this questionnaire indicate better QOL.

### Piano training program

A 4-month long piano training program was specifically designed and implemented for elderly people by a professional music teacher and pianist (Heidi Figueroa). The experimental group of 25 participants was divided into two groups with the intention of creating classes of 13 participants maximum. These allowed to create a participate environment were more personalized attention could be received by the student. Both classes received the same piano instruction.

Group piano lessons, lasting one hour and a half, were given in a community-center on a weekly basis by the same music teacher who had designed the program. Classes combined essential theoretical knowledge about music notation and theory with actual practice of piano playing. Three homework exercises requiring the playing of a piano sequences were given in each lesson, and participants were committed to practice independently at least 45 min per day at least 5 days per week (~4 h per week). They were given a calendar to register daily their devoted time. Each day they had to practice playing a piano sequence 10 times with their dominant hand, and 10 more times with their non-dominant hand. Subjects had free access to practice the piano during all the week in the community-center. However, many of the participants decided to buy a portable piano keyboard to practice at home.

In each face-to-face lesson, participants had to play the piano sequence that they had practiced during the week before the class. This methodology was used to motivate participants with a weekly goal (playing in front of the class) and also so that other classmates heard feedback and learned from the errors of their partners. Finally, at the end of the class each participant had to practice and solve doubts about the new exercises proposed for that week. The level of difficulty of the piano program increased gradually. The piano learning phases and their increase in difficulty are specified in Table 2.

**Table 2:** **Piano learning phases and their gradual increase in difficulty**.

**Piano learning phases**	**Piano exercises**
1st Phase	Participants practiced ascendant and descendent progressions of consecutive musical notes with the five fingers of the hand. Each exercise was repeated with the right hand in the treble clef, and with the left hand in bass clef. Finally, subjects had to practice with both hands together.
2nd Phase	Alternating musical notes were practiced with the five fingers of both hands. Ascendant and descendent chord triads were practiced increasing the distance between musical intervals.
3rd Phase	Practice of “thumb under” exercise in the piano, with both hands.
4th Phase	Playing different melodies with each hand and alternating.
5th Phase	Playing a melody with the right hand, while playing long musical notes with the left hand.
6th Phase	Playing a melody with the left hand, while playing long musical notes with the right hand.
7th Phase	Playing two different melodies at the same time by alternating learned movements with the hands.
8th Phase	Adding articulation, staccato, and legato.
9th Phase	Adding indications of expression.

### Statistical analysis

Demographic data, pre-program scores, and mean days of practice per week were analyzed by running independent *t*-test between the two groups (piano and control) when the data was normally distributed and Mann–Whitney test when not normally distributed. This was done in order to ensure that both groups did not differ in age, education, baseline scores, and in the frequency they trained each kind of activity.

In order to identify possible changes associated to the implementation of the piano program the analysis of data was carried out using 2-Group × 2-Condition Split-Plot Analysis of Variance (ANOVAs). The between-subjects factor in the analysis was Group which included two levels, subjects that were assigned to the piano group or control group (those that did not take piano lessons and did other type of leisure activities). The within-subjects factor was Condition with two levels, pre-program and post-program (before the piano lessons vs. 4 months later, after the piano lessons; same interval for control group). Significant effects were considered at a 95% level of confidence; however some trends will be mentioned in the report due to the relatively small sample size of the study (*n* = 16 subjects in the control group and *n* = 13 in the piano group).

## Results

Two blocks of tests, one for cognitive domains (motor ability, attention, and executive function) and the second for mood and QOL, were carried out in a group of older adults (61–84 years old; *n* = 13) that for 4 months received piano classes and practiced for 45 min/day against a control group (63–80 years old; *n* = 16) that had done other kind of leisure activities. We have found significant improvements following the piano training in some tests that measure attention and executive function (Stroop and a trend in TMT-A). Moreover, we also observe a significant improvement in affective states (BDI and POMS) and some domains of QOL (WHOQOL-BREF).

### Demographic variables, frequency of training, and pre-program scores

We did not find significant differences in the age, years of education, estimated intelligence as measured with WAT, MMSE, and FAB between the two groups. Furthermore, concerning the frequency of training, no significant differences were found in the mean days of practice between the piano group and the leisure activities performed by the control group. The mean minutes of piano training per day carried out by subjects in the experimental group was 21.15 (*SD* = 9.73). We do not have the equivalent information (time of dedication to leisure activities per day) for the control group. Table 3 summarizes means and standard deviations of the screening tests, demographic variables, and training days per week.

**Table 3:** **Means ± *SD* of screening tests, demographic variables, and days trained per week**.

	**Experimental group**	**Control group**
Age	69.30 ± 2.03	69.56 ± 1.43
Years of education	15.38 ± 4.79	13.38 ± 6.09
MMSE	29.31 ± 1.32	29.38 ± 0.96
FAB	17.69 ± 0.48	16.94 ± 1.18
WAT	107.14 ± 3.82	107.33 ± 7.09
Mean training days	4.85 ± 1.68	4.06 ± 1.48

In the pre-program scores we found a significant difference between groups in DSF (Mean number digits and *SE* in piano group: 8.77 ± 0.59; Mean number digits and *SE* in control group: 7.25 ± 0.41; *p* = 0.04) and Digits Span Backwards (Mean number digits and *SE* in piano group: 6.00 ± 0.36; Mean number digits and *SE* in control group: 4.63 ± 0.41; *p* = 0.02), but not in Digits Amplitude Backward and Forward. The implications of this difference will be discussed in the latter section. No other significant differences were found between groups on pre-program scores (baseline).

### Motor ability, attention, and executive function

**Figure 1:**
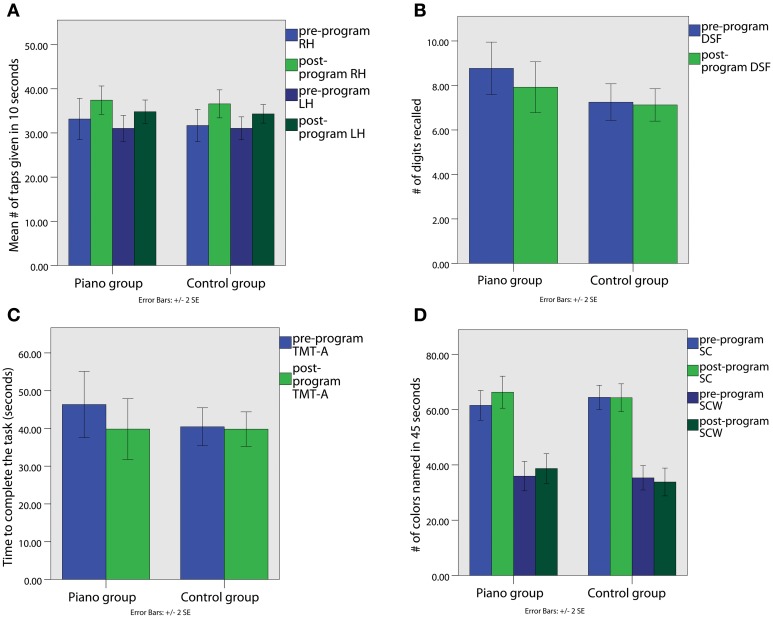
**(A)** Performance in Finger Tapping Test with Right Hand (RH) and Left Hand (LH); **(B)** Performance in Digit Span Forward (DSF); **(C)** Performance in Trial Making Test Part A (TMT-A); **(D)** Performance in Stroop Color (SC) and Stroop Color-Word (SCW).

In the FTT, there was a significant main effect of Condition (before the piano lessons vs. 4 months later) with RH, *F*_(20.36)_, *p* = < 0.001, η^2^_*p*_ = 0.43, and LH, *F*_(39.46)_, *p* < 0.001, η^2^_*p*_ = 0.59. Interestingly, a significant improvement in the finger tapping both for LH and RH occurred not only in the piano group (RH pre-program Mean number taps and *SE*: 33.16 ± 2.33; RH post-program Mean number taps and *SE*: 37.43 ± 1.60; LH pre-program Mean number taps and *SE*: 31.70 ± 1.82; LH post-program Mean number taps and *SE*: 36.59 ± 1.58) but also in the control group (RH pre-program Mean number taps and *SE*: 31.70 ± 1.82; RH post-program Mean number taps and *SE*: 36.59 ± 1.58; LH pre-program Mean number taps and *SE*: 31.05 ± 1.38; LH post-program Mean number taps and *SE*: 34.33 ± 1.03). Figure 1A indicates that the number of finger taps in a given time interval tends to increase over time for both groups. No significant differences were found in the Grooved Pegboard and Blocks Design tests.

Some tests such as DSF showed significant differences in the pre-program vs. post-program assessment in the piano but not in the control group. Repeated measures ANOVA showed a significant main effect for Condition in DSF, *F*_(5.81)_, *p* = 0.023, η^2^_*p*_ = 0.18, indicating that the number of digits recalled by the piano group decreased from pre-program (Mean number digits and *SE*: 8.77 ± 0.59) to post-program assessment (Mean number digits and *SE*: 7.92 ± 0.57; Figure 1B). No change was observed in the control group (pre-program Mean number digits and *SE*: 7.25 ± 0.41; post-program Mean number digits and *SE*: 7.12 ± 0.36). The possible causes for these changes will be discussed below. Moreover, no significant effects were found for Digits Span Backwards, Digits Amplitude Forward and Digits Amplitude Backwards. Moreover, no significant differences were found in Spatial Span.

A significant main effect of Condition was found on the TMT-A [*F*_(5.97)_, *p* = 0.022, η^2^_*p*_ = 0.19]. The improvement in the test was almost significantly larger in the piano group, *F*_(4.06)_, *p* = 0.054, η^2^_*p*_ = 0.14. As illustrated in Figure 1C, the time to complete the TMT-A task in the piano group decreased from the pre-program (Mean = 46.33″; *SD* = 4.28) to the post-program assessment (Mean and *SE*: 39.83″ ± 4.02), while it did not differ in the control group (pre-program Mean and *SE*: 40.33″ ± 2.52; post-program Mean and *SE*: 39.81″ ± 2.28). Furthermore, no significant effects were found for the TMT-B.

Regarding the Stroop test, we found a significant main effect of Condition [*F*_(4.98)_, *p* = 0.034, η^2^_*p*_ = 0.16], and Group × Condition interaction [*F*_(5.51)_, *p* = 0.027, η^2^_*p*_ = 0.17] in the SC subtest. As shown in Figure 1D, the scores of subjects in the piano group increased in the post-program (Mean number words and *SE*: 66.54 ± 2.82) compared to pre-program assessment (Mean number words and *SE*: 61.54 ± 2.69), corresponding to the number of colors named during 45 s. The control group did not show such a change over time (pre-program Mean number words and *SE*: 64.44 ± 2.19; post-program Mean number words and *SE*: 64.31 ± 2.50). Additionally, a significant main effect in Group × Condition interaction for the SCW subtest that measures executive function and cognitive inhibition was found, *F*_(4.54)_, *p* = 0.042, η^2^_*p*_ = 0.54. As shown in Figure 1D, the piano group increased their score from pre-program (Mean number words and *SE*: 36.08 ± 2.60) to post-program assessment (Mean number words and *SE*: 38.69 ± 2.68), while the control group performance decreased over time (pre-program Mean number words and *SE*: 35.31 ± 2.20; post-program Mean number words and *SE*: 33.81 ± 2.52). No significant effects were found in the SW subtest.

Finally, non-significant main effects were found for SDMT and Lexical Task. Table 4 summarizes means and Standard Errors (*SE*) for all test included to measure motor ability, attention and executive function.

**Table 4:** **Means (*SE*) for all test that measure attention, executive function, and motor ability**.

**Measures**	**Experimental group**		**Control group**	
	**Pre-test**	**Post-test**	**Pre-test**	**Post-test**
Finger tapping (right hand)	33.16 (2.33)	37.43 (1.60)	31.70 (1.82)	36.59 (1.58)
Finger tapping (left hand)	31.05 (1.46)	34.82 (1.32)	31.05 (1.30)	34.33 (1.05)
Grooved pegboard (right hand)	76.61 (2.38)	73.62 (2.82)	72.78 (3.16)	73.75 (2.82)
Grooved pegboard (left hand)	82.29 (3.97)	82.77 (3.11)	78.00 (3.89)	83.19 (3.49)
Cubes	31.53 (2.22)	33.84 (2.01)	32.81 (2.62)	33.13 (2.53)
Digits span forward	8.77 (0.59)	7.92 (0.57)	7.25 (0.41)	7.12 (0.36)
Digits amplitude forward	5.77 (0.28)	5.38 (0.35)	5.13 (0.24)	5.13 (0.24)
Digits span backwards	6.00 (0.36)	5.92 (0.50)	4.63 (0.41)	4.69 (0.46)
Digits amplitude backwards	4.46 (0.22)	4.46 (0.24)	3.81 (0.26)	3.81 (0.26)
Corsi span forward	6.77 (0.41)	6.92 (0.49)	5.94 (0.35)	6.75 (0.31)
Corsi amplitude forward	5.00 (0.25)	5.07 (0.31)	4.56 (0.26)	4.75 (0.27)
Corsi span backwards	6.38 (0.40)	6.38 (0.37)	6.06 (0.32)	6.13 (0.35)
Corsi amplitude backwards	4.69 (0.29)	4.54 (0.22)	4.50 (0.22)	4.50 (0.22)
TMT part A	46.33 (4.38)	39.83 (4.02)	40.44 (2.52)	39.81 (2.28)
TMT part B	98.23 (16.85)	90.38 (8.07)	90.06 (12.65)	109.50 (15.22)
SDMT	42.15 (3.45)	44.31 (3.62)	37.94 (2.99)	37.81 (2.88)
Stroop-word	99.77 (4.85)	101.62 (4.20)	101.94 (2.12)	102.25 (1.88)
Stroop-color	61.54 (2.69)	66.54 (2.82)	64.44 (2.19)	64.31 (2.50)
Stroop-color word	35.92 (2.66)	38.69 (2.68)	35.31 (2.20)	33.81 (2.52)
Lexical task	14.31 (1.21)	13.92 (0.89)	14.94 (1.10)	13.31 (1.29)

### Mood and quality of life

**Figure 2:**
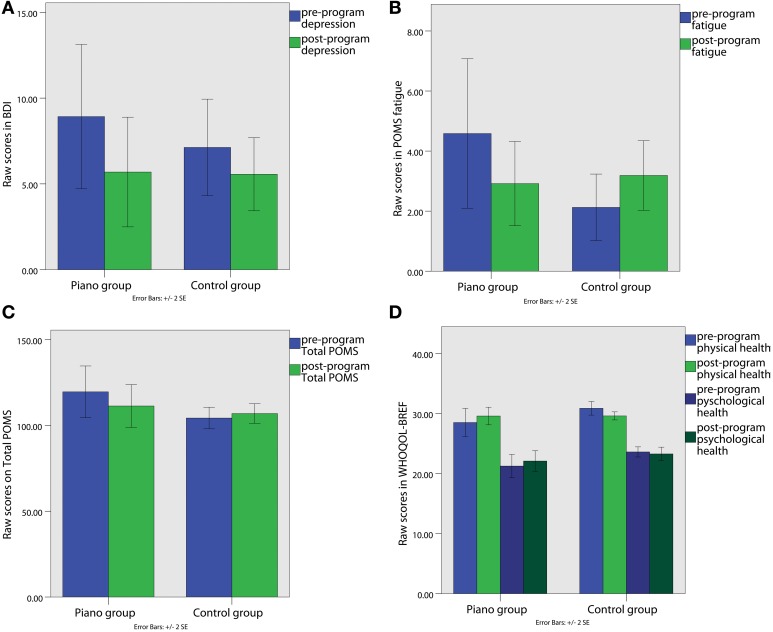
**(A)** Beck Depression Inventory (BDI) scores; **(B)** Fatigue sub-scale raw score of the POMS; **(C)** Profile of Mood States (POMS) total raw score; **(D)** WHOQOL-BREF raw scores in the physical health and psychological health domains.

We found that BDI scores drop from the pre-program to the post-program assessment in both piano (pre-program Mean score and *SE*: 8.92 ± 2.10; post-program Mean score and *SE*: 5.69 ± 1.60) and control groups (pre-program Mean score and *SE*: 7.13 ± 1.41; post-program Mean score and *SE*: 5.56 ± 1.06). A significant main effect for Condition was found in the BDI, *F*_(7.36)_, *p* = 0.012, η^2^_*p*_ = 0.21, as can be seen in Figure 2A.

In the POMS there was a significant Group × Condition interaction in the Fatigue factor [*F*_(6.86)_, p=0.015, η_*p*2_ = 0.20] and in the total POMS score [*F*_(4.91)_, *p* = 0.036, η^2^_*p*_ = 0.16]. Figures 2B,C, indicate that the fatigue scores (pre-program Mean score and *SE*: 4.23 ± 1.20; post-program Mean score and *SE*: 2.92 ± 0.70) and the total score in the POMS (pre-program Mean score and *SE*: 117.70 ± 7.18; post-program Mean score and *SE*: 111.33 ± 6.23), related to psychological distress, decreased from the pre-program to the post-program assessment in the piano group. However, the control group shows exactly the opposite pattern, since the scores in the total score (pre-program Mean score and *SE*: 104.31 ± 3.14; post-program Mean score and *SE*: 106.93 ± 2.85) and fatigue (pre-program Mean score and *SE*: 2.13 ± 0.55; post-program Mean score and *SE*: 3.19 ± 0.58) seemed to increase over time.

In the WHOQOL-BREF we found a significant Group × Condition interaction in the Physical Health [*F*_(6.75)_, *p* = 0.015, η^2^_*p*_ = 0.20] and in the Psychological Health domains [*F*_(4.45)_, *p* = 0.045, η^2^_*p*_ = 0.151). As shown in Figure 2D, while the physical (pre-program Mean score and *SE*: 28.85 ± 1.13; post-program Mean score and *SE*: 29.85 ± 0.72) and psychological health scores (pre-program Mean score and *SE*: 30.81 ± 0.53; post-program Mean score and *SE*: 29.50 ± 0.33) increased in the post-program in comparison to the pre-program assessment in the piano group, the scores of the control group show a tendency to decrease or maintain the same in physical (pre-program Mean score and *SE*: 30.81 ± 0.53; post-program Mean score and *SE*: 29.50 ± 0.33) and psychological domains (pre-program Mean score and *SE*: 23.50 ± 0.41; post-program Mean score and *SE*: 23.27 ± 0.56). Moreover, no significant effects were found in Social and Environmental Health factors. Table 5 summarizes means and *SE* in the mood and QOL questionnaires.

**Table 5:** **Mean (*SE*) for the mood and quality of life questionnaires**.

**Measures**	**Experimental group**		**Control group**	
	**Pre-test**	**Post-test**	**Pre-test**	**Post-test**
Beck depression inventory	8.92 (2.10)	5.69 (1.60)	7.13 (1.41)	5.56 (1.06)
POMS tension	7.23 (1.38)	5.46 (0.82)	5.25 (0.87)	5.50 (0.77)
POMS depression	8.23 (2.88)	6.62 (2.65)	3.25 (0.67)	4.50 (0.90)
POMS anger	9.69 (1.99)	7.69 (1.43)	5.69 (0.79)	5.63 (0.66)
POMS vigor	16.61 (1.38)	16.38 (1.58)	16.56 (1.23)	16.75 (1.07)
POMS fatigue	4.23 (1.20)	2.92 (0.70)	2.13 (0.55)	3.19 (0.58)
POMS confusion	4.92 (0.86)	4.08 (0.61)	4.56 (0.65)	4.88 (0.71)
POMS total score	117.70 (7.18)	111.33 (6.23)	104.31 (3.14)	106.93 (2.85)
WHOQOL physical health	28.85 (1.13)	29.85 (0.72)	30.81 (0.53)	29.50 (0.33)
WHOQOL psychological health	21.61 (0.96)	22.08 (0.86)	23.50 (0.41)	23.27 (0.56)
WHOQOL social health	10.85 (0.74)	11.33 (0.63)	11.88 (0.31)	12.00 (0.41)
WHOQOL environmental health	30.92 (1.20)	32.00 (1.13)	33.06 (0.82)	33.27 (0.77)

## Discussion

The main goal of this study was to assess the effect of 4 months piano lessons on cognitive function, affective states, and QOL of older adults. Our control group was not passive, but participated in other type of leisure activities, allowing us to detect changes in different parameters that could be specific to musical training and not just to the fact of being involved in some stimulating activity with social interactions. We found significant effects in cognitive abilities related to attention and executive function (significant improvement in Stroop and a positive trend in TMT-A). Moreover, we also observed a significant improvement in some domains of affective states (BDI and POMS) and QOL (WHOQOL-BREF). On the one hand, the hypothesis that the piano learners would increase their performance in cognitive domains was partially supported by the improved performance in some tests. On the other hand, the hypothesis that there would be an improvement in mood, subjective well-being, and QOL was more strongly, but also partially, supported.

Our piano learning program for 4-months resulted in an improvement in the Stroop Test (SC and SCW), reflecting an enhancement of selective processing, automaticity, and inhibitory control. This improvement seems to be specifically caused by the musical training since the control group did not show such a pattern change through time. Interestingly, a previous study showed that professional musicians had significantly smaller color-word interference effects in the Stroop task (Travis et al., 2011). Moreover, our musical instrument training program also seemed to enhance visuomotor tracking, attention, processing speed, and motor function, as can be seen on an almost significant trend in the TMT-A.

Between our experimental and control group there were not the significant differences in TMT-B and SDMT that Bugos et al. (2007) found in their individualized piano instruction program. We speculate that this might be due to the shorter duration of our training regime, since there is evidence for an improvement of executive function reflected by the Stroop Test. Further studies should explore the specific effect of piano training in different stages of the learning process to better understand which cognitive capacities can improve with these types of programs and in which time periods they occur. Paradoxically, we found a small but significant decrease in performance in the digit span forward test, but not for the digits span backwards test which requires a higher cognitive demands since it includes item manipulation and not only mere storage (Reynolds, 1997). Taking into account that the normal range of digits forward for old people is 5 ± 1 (Peña-Casanova et al., 2009a), it should be noticed that the scores during the post-test (Mean and *SD*: 7.92 ± 0.57) were still within the normal ranges despite the fact that there was a decrease in performance (of approximately one sequence) (Myerson et al., 2003). However, this result should be interpreted with caution since Digits Span is the only measure where there were significant differences between the groups at the baseline.

Concerning motor abilities, we found that the piano group as well as the control group improved on a finger tapping task. We speculate that this significant change in manual dexterity for both groups could be due to practice effects of the test. Beglinger et al. (2005) found a large improvement in finger tapping test from a first session to a second session in a control group, although this difference was not maintained in a third and fourth trial. However, changes observed in this motor test could be also explained by the fact that the control group participated in physical activities (e.g., gymnastics, yoga, walking, Tai chi, dancing) involving the motor system. Some studies suggest that physical exercise can have an influence on the performance of these measures (Blumenthal et al., 1991; Dash and Telles, 1999). Based on the design of this study, and the heterogeneity of the control group, we cannot elucidate whether this increase in motor performance through time was caused by practice of the test or by the different trained activities.

Overall, our results indicate that learning to read musical notation and playing the piano may enhance mood and certain aspects of the QOL in older adults. Specifically, both groups showed a significant decrease in depression symptoms. Past evidence suggests that an active lifestyle and participation in leisure activities is related to lower depression rates (Dupuis and Smale, 1995). Nevertheless, piano training seemed to have additional emotional benefits over other types of leisure activities. A significant increase in some aspects of QOL, related to psychological well-being and physical health, were found. Moreover, this was further corroborated by the fact that measures associated to psychological distress and fatigue decreased in the piano group, but not in the control group. Depression and psychological distress are highly prevalent symptoms in latter stages of life and have been shown to be related to an increase risk of dementia and cardiovascular diseases (Saczynski et al., 2010; Henderson et al., 2013). Based on these outcomes, music learning and group piano training could be an effective intervention toward battling depression and promoting a positive mood in older adults.

A series of follow-up studies have shown that participation in leisure activities in later-life (e.g., reading, writing, crossword puzzles, board or card games, group discussions, among others), especially intellectual activities, is related to increase in CR (Scarmeas and Stern, 2003). Based on the evidence that brain plasticity occurs in older adults (Boyke et al., 2008), it is suggested that lifestyle could make brain processing more efficient by using pre-existing resources or by activating compensatory mechanisms (Stern, 2002). However, the extent to which different leisure activities may contribute to promote CR and preserve cognitive abilities is not well-understood. In this study, we have shown that specific motor, attention and memory skills acquired through piano training might transfer to an improvement in other type of tasks as assessed by a trend in TMT-A and a significant effect in the Stroop Test. We hypothesized that this specific-skill transferability of music learning to other domains is related to the unique multimodal nature of learning a musical instrument (Bugos et al., 2007). Moreover, as Green and Bavelier (2008) suggested, the fact that the training program corresponds to a real life experience and is not a simple laboratory manipulation may have played a role in the skill-transfer effects. Based on our results, we suggest that learning to play piano in older ages might contribute to promote CR and improve or maintain cognitive function in later stages of life.

We will next discuss some of the limitations of our study. The first is related to the relatively small sample size (*N* = 29), in part due to the number of participants (*N* = 12) that had to be excluded for different reasons (see section Participants). Second, in this study we did not assign participants randomly to the groups, but first the experimental group was recruited based on voluntary commitment and next the control age-matched active group was recruited. We cannot rule out the possibility that the results were influenced by predispositions of participants in the piano group. Besides, the psychologist in charge of the carrying out the neuropsychological assessments was not blind to the membership of individuals in the two groups. This could have had some undesired impact, although all the assessments were carried out in a very systematic and neutral way. Future studies should explore the effects of music training with bigger sample sizes, random assignment to the group, and blinded examiners, to explore the generalizability of results. Third, the group class format of the piano training makes it difficult to elucidate whether some of the observed effects were also related to social interactions in the weekly class, although the daily piano practice was individual. At least part of the control group leisure activities had also a group format, so that the possible effect of the format was partially controlled. The fourth limitation is derived from the time invested by each participant in practicing the piano. We instructed subjects to practice for 45 min per day, but we cannot rule out the possibility that some students practiced more or less time. In this sense, we also do not have exact information of how much time per day each participant in the control group practiced their leisure activities. So we cannot exclude the possibility that the results are a consequence of the amount of training per day and not by the type of training. However, this is highly improbable since no significant differences existed between the amounts of days that subjects practiced per week. Finally, all older adults that participated in this study had an active lifestyle reflected by their involvement in leisure activities before the start of this investigation. It would be interesting if future research studied the effects of musical learning and other training regimes in subjects' naïve to leisure activities to observe whether changes observed are similar or different with respect to this issue.

This study provides evidence that piano lessons can have a positive impact on certain domains of cognitive function, mood and QOL of older adults. Learning to play the piano is a multi-modal and complex activity that requires learning to map visual information from the musical notation to a motor response that produces a sound. Furthermore, playing the piano is also a motivating activity that allows the learner to have a constant and immediate auditory feedback of his performance. Finally, group piano lessons are an accessible and affordable leisure activity, which can easily be offered for example in community centers. Based on these advantages we propose piano lessons as an enriching and inspiring activity that can contribute to successful aging.

### Conflict of interest statement

The authors declare that the research was conducted in the absence of any commercial or financial relationships that could be construed as a potential conflict of interest.
